# Biomarkers of cellular aging during a controlled human malaria infection

**DOI:** 10.1038/s41598-021-97985-y

**Published:** 2021-09-21

**Authors:** Aurelie Miglar, Isaie J. Reuling, Xi Zen Yap, Anna Färnert, Robert W. Sauerwein, Muhammad Asghar

**Affiliations:** 1 Division of Infectious Diseases, Department of Medicine Solna, Karolinska Institutet, Stockholm, Sweden; 2 Department of Infectious Diseases, Karolinska University Hospital, Stockholm, Sweden; 3 Center for Molecular Medicine, Karolinska Institutet, Stockholm, Sweden; 4 Department of Microbiology, Radboud University Medical Center, Geert Grooteplein 28, Microbiology 268, 6500 HB Nijmegen, The Netherlands; 5 TropIQ Health Sciences, Transistorweg 5-C02, 6534 AT Nijmegen, The Netherlands

**Keywords:** Infectious diseases, Parasitology, Predictive markers, Experimental models of disease

## Abstract

Cellular aging is difficult to study in individuals with natural infection, given the diversity of symptom duration and clinical presentation, and the high interference of aging-related processes with host and environmental factors. To address this challenge, we took advantage of the controlled human malaria infection (CHMI) model. This approach allowed us to characterize the relationship among cellular aging markers prior, during and post malaria pathophysiology in humans, controlling for infection dose, individual heterogeneity, previous exposure and co-infections. We demonstrate that already low levels of *Plasmodium falciparum* impact cellular aging by inducing high levels of inflammation and redox-imbalance; and that cellular senescence reversed after treatment and parasite clearance. This study provides insights into the complex relationship of telomere length, cellular senescence, telomerase expression and aging-related processes during a single malaria infection.

## Introduction

Cellular aging, the decline in the function, growth and division of cells is believed to be involved in the onset of various age-related diseases. Previous research suggests that infections are possible key-drivers of accelerated aging^1,2^, including malaria, which remains a significant health problem and led to more than 229 million cases and 409,000 reported deaths worldwide in 2019^3^. The clinical consequences of acute and chronic malaria are well recognized, however, there might be a hidden long term cost for the host. Studies in birds have shown that mild chronic malaria infections accelerate telomere shortening, leading to decreased fitness and reduced life span^4,5^. A follow up study in travelers with imported malaria showed that an acute *Plasmodium falciparum* infection also affects cellular aging markers in humans^1^. While acute malaria infection has a pronounced effect on cellular aging, it remains elusive whether an extremely low parasitic *P. falciparum* infection is proficient to impact cellular aging kinetics.

Cellular aging is defined as progressive loss of physiological integrity and thus, the driving intrinsic component of increased age-specific mortality^6^. There are several hallmarks of cellular aging including telomere length (TL), cellular senescence and telomerase activity^7^. Telomeres, the non-coding repetitive sequences, play an essential role in chromosomal integrity, decrease with age and are linked to cellular senescence^8,9^. The effect of telomere shortening can be counteracted by the reverse transcriptase, telomerase; an enzyme expressed primarily in stem and germ cells^10^. Existing studies have linked accelerated telomere shortening to several human pathologies^11–13^. Besides malaria, shorter telomeres were shown in patients with HIV, Epstein–Barr virus (EBV), chronic hepatitis-C virus (HCV) and cytomegalovirus (CMV)^14–17^; with significant correlation of HCV and CMV to changes in the expression of the cellular senescent marker CDKN2A/p16INK4a^1,18,19^.

Asexual *P. falciparum* parasitaemia triggers an array of host responses in both innate and adaptive immune system, including the expansion of helper and effector CD4^+^ T cells, governing the cell-mediated immune response through the production of pro-inflammatory cytokines (IFN-γ, TNF-α, IL-1β IL-12 and IL-18)^20,21^, and generating free radicals to clear parasites. Inflammation and oxidative stress are further involved in age-related conditions^22^, by inducing age-associated functional losses^23^. However, the exact mechanisms of oxidative stress induced aging and its correlation to cellular senescence (CDKN2A/p16INK4a) is still not clear.

The study of aging markers in natural infections is challenging, as our knowledge is limited about whether accelerated aging is a direct consequence of infection, or whether aging markers increase susceptibility to disease. One previous study in human volunteers showed that individuals with longer telomeres were less prone to developing symptoms when infected with a respiratory virus, however aging markers were not followed over time after infection^24^. Moreover, the majority of studies in human are cross-sectional or cohort studies in individuals with acute or chronic infections with various clinical presentations and infection duration^25^. Hence, studying cellular aging kinetics in disease optimally requires a sample prior to infection and a well-controlled study setting.

Over the past decade, controlled human malaria infection (CHMI) has become a highly valuable model to study malaria pathophysiology and immunology, providing an efficient way to test the efficacy of new drugs and vaccines^26^. Here we used a CHMI, where healthy volunteers were infected with *Plasmodium falciparum* parasites using a standardized protocol with a known inoculation dose, well-defined pre and post infection follow up time points, close monitoring and prompt treatment upon the onset of symptoms and/or detection of parasite levels above protocol standards to examine the effect of low level parasitaemia on cellular aging. We characterize cellular aging markers together with a wide panel of inflammatory cytokines and anti-oxidant gene expression, blood biochemical analysis before, during and after infection, and further evaluate aging-associated loss of function, using biochemical liver function assays.

## Methods

### Study design

The CHMI study was conducted at the Radboud University Medical Center, Nijmegen, the Netherlands^26^. The Central Committee for Research involving human subjects (CCMO) located in the Netherlands, and the Western Institutional Review Board (WIRB) located in the USA approved the study protocol for the trial. Informed consent was obtained from all study participants. The study procedure corresponds to principles expressed in the Declaration of Helsinki and Good Clinical Practice standards and is registered in the database Clinical Trials.gov (NCT02836002), and all applied methods were carried out in accordance with relevant guidelines and regulations. The malaria challenge study recruited 16 healthy, malaria-naïve Dutch individuals from June until November 2016, and was conducted pursuant to the standard CHMI described in Sauerwein et al.^27^. Majority of the study participants were female (75%), age ranged from 20 to 29 years (median = 22), and bodyweight ranged from 57 to 90 kg (median = 73). General and baseline characteristics of the CHMI participants are described elsewhere^26^.

### CHMI procedure

**Figure 1:**
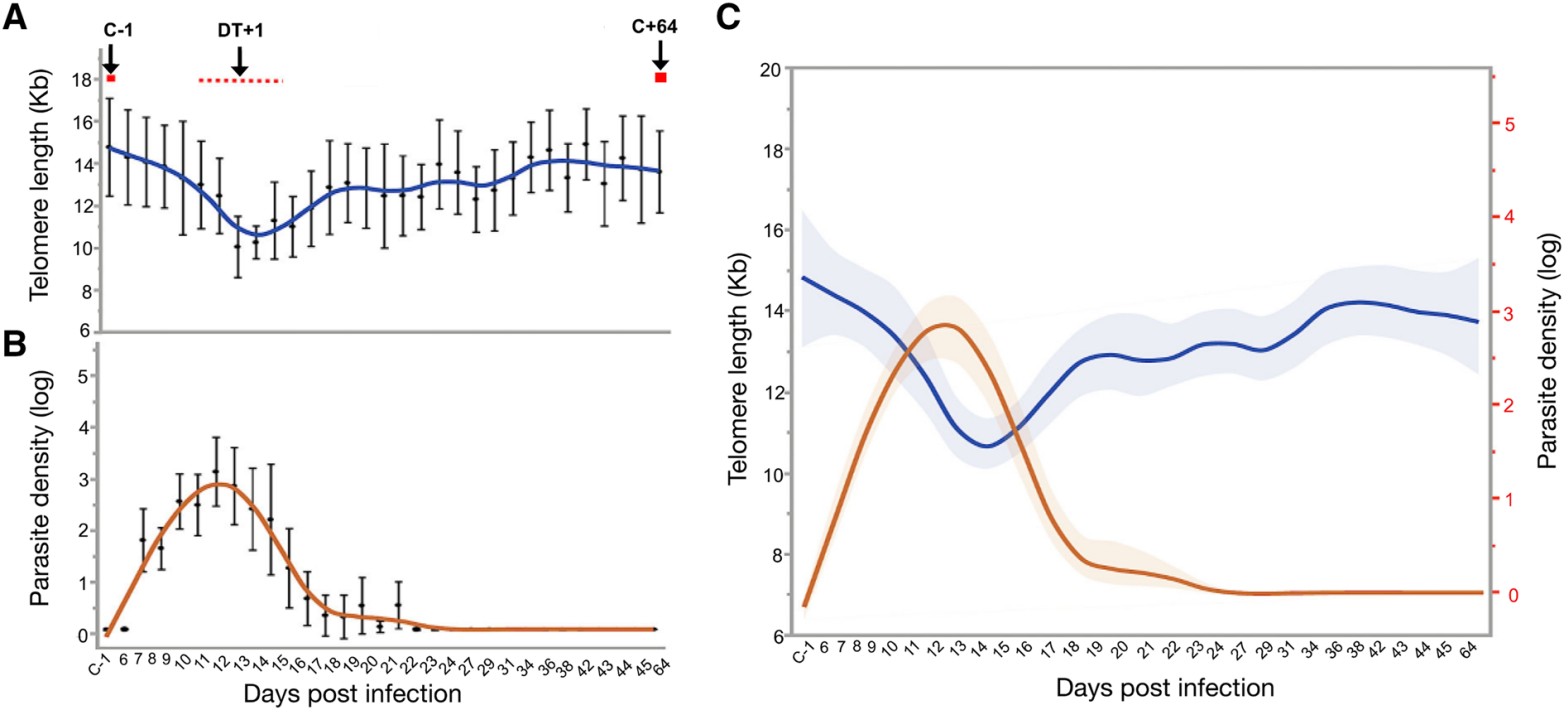
Telomere length and *P. falciparum* parasite densities before, during and after a controlled human malaria infection (CHMI). (**A**) Telomere length dynamics in kilobase pairs (kb) on respective study days of malaria infection. Arrows indicate the study time point prior infection (C-1, base line sample), day post treatment (DT+1), and time of end treatment/end of study (C+64). (**B**) Log values of *P. falciparum* parasite density (*Pf*/mL blood) during malaria infection. Peak parasite density was observed during day 11–12, and ranged from 2435 to 197,500 *Pf*/mL (median 22,092 *Pf*/mL), corresponding to 0.000062–0.0049% infected red blood cells (iRBC; median 0.00055%). Solid blue and orange lines represent predicted means, with black dots representing the detected mean value at each time point. Error bars denote the 95% CI of the mean. (**C**) Relationship between telomere length (kb) and parasite density (log values) during malaria infection. Solid blue line represents predicted mean of telomere length, solid orange line represents predicted mean of parasite, with shaded areas denoting the confidence of the mean, (N = 16, observations _TL_ = 481, observations _parsitaemia_ = 278).

Participants were one-time exposed to bites of five *P. falciparum* (*Pf*) infected mosquitoes for 10 min each, and monitored twice daily upon day six post infection (PI). Upon parasite density reaching ≥ 5000 parasites per milliliter blood (*Pf*/mL; 0.0001% iRBC), the initial sub-curative treatment of 500 mg/25 mg sulfadoxine–pyrimethamine (Roche, Boulogne-Billancourt, FR) or 480 mg of piperaquine phosphate (PCI Pharma Services, Tredegar, UK) was administered and participants were monitored twice daily for four days. Consequent to parasite density reaching ≥ 1500 *Pf*/mL (0.00004% iRBC), the second treatment was administered, and monitoring was pursued daily for three days, followed by three times a week until final treatment with curative regiment (ET) of atovaquone/proguanil on day 42. Study participants were followed until day 64 post infection and blood samples were collected on every respective visit to monitor the development of parasite density and blood safety parameters (see Fig. 1). No serious adverse events were reported during the study period. Detailed description of the study procedure can be found in Reuling et al.^26^.

Parasitological assessment was performed by microscopy of thick blood smears and quantitative real-time PCR (qPCR)^28,29^. Parasite density evaluated by qPCR in *Pf*/mL blood (*Pf*/mL) was used in our analysis, and was interconverted to percentage of infected red blood cells (% iRBC).

An overview of the number of study participants and time-points, and the measured markers per individual are found in supplementary materials (Table S2).

### Nucleic acid extraction

Total nucleic acid was extracted from in total 481 whole blood samples from the respective 29–31 time-points in 16 study participants, using an automated extraction system (MagNA Pure LC Total Nucleic Acid Isolation Kit, Roche Diagnostics).

### Telomere measurement

Telomere length was assessed by quantitative real-time PCR (qPCR) by following the method described in Asghar et al.^1^. This approach was chosen in respect of the large number of samples (N = 481), considering the time consumption of other quantitative measurements (TRF and STELA). DNA was purified from total nucleic acid, using Ambion RNase I (cat # AM2295; Thermo Fisher Scientific), and diluted to a concentration of 2 ng μl^−1^ to measure TL on a QuantStudio 5 qPCR instrument. A telomere-specific amplicon set of primers (*tel1*, *tel2*) was used, together with a single-copy gene amplicon set of primers (*HBG1*, *HBG2*) to control for total amount of DNA in each reaction^30^. The total qPCR reaction contained 25 μl, including 5 μl DNA (2 ng/μl), 10 μl Supermix (PowerUp SYBR Green Master Mix; Applied Biosystems, cat # A25778; Thermo Fisher Scientific), 0.2 μl of each *tel* and *HBG* primer (10 μmol/L) and ddH_2_O. The primer sequences for *tel* are found in O’Callaghan and Fenech^36^ and sequences of single-copy gene primers are described elsewhere^30,31^. Each plate included samples, a Gold standard (an arbitrary sample included on each plate to control for inter-plate variability), a serial dilution of sample (to produce standard curve), and a non-template control. All samples were run in duplicates for each primer set, and each primer set was run on a separate plate. Average quantitative values of duplicates were normalized across the plates by dividing them by the plate value of the Gold control to avoid inter-plate variability and to obtain plate adjusted amounts of telomere and single-copy gene sequence. Relative TL was calculated by dividing the normalized telomere sequence (T) by normalized single copy gene sequence (S). This approach corresponds to ΔΔ*C*_*T*_ values, as – log2 of T/S is equal to ΔΔ*C*_*T*_^30,32^. Initially, our samples were run to analyze T/S ratio, while a genomic DNA sample (515 kb; #8918 Science Cell) was included as an additional reference sample on later analysis of 92 samples to compare T/S values with kb values. Our T/S ratio was then extrapolated for each sample based on the formula (T/S ratio 1 = 538 bp), to calculate TL in kb for all samples, referring to our human genomic DNA reference sample. Inclusion of gDNA sample on every qPCR plate (N = 24) resulted in non-significant variation in quantitative values (Miglar et al. unpublished). Moreover, our method showed high intra- and inter-plate repeatability, ICC > 0.98, which was in line with our previous studies^1,4,33^.

### RNA purification and cDNA preparation

RNA was isolated from whole blood as total nucleic acid at three time-points C-1 (pre-infection), DT+1 (day after treatment 1) and C+64 (day 64 post-infection) for all study participants, with one participant lacking the sample for C+64. Total nucleic acid was treated with DNase to remove DNA contamination, using the TURBO DNA-free Kit (cat # AM1907; Invitrogen, Thermo Fisher Scientific). After RNA assessment, 100–150 ng RNA was converted to cDNA applying the SuperScript VILO cDNA Synthesis Kit (cat # 11754250; Invitrogen, Thermo Fisher Scientific), according to the manufacturer´s instructions.

### Gene expression

Real-time PCR was performed on the corresponding cDNA synthesized from samples C-1, DT+1 and C+64. Relative gene expression of CDKN2A, telomerase and anti-oxidants was determined using the comparative Δ*C*_*T*_ method by calculating the *C*_*T*_ values of the target gene against the *C*_*T*_ values of our reference gene (GAPDH)^1^. Our target gene and endogenous control (GAPDH) were amplified in same wells, run in duplicates and respective *CR*_*T*_ values were averaged before performing the Δ*C*_*T*_ calculation (Δ*C*_*T*_ = *C*_*T*__Target_ – *C*_*T*__GAPDH_). The corresponding ratio refers to “expression of gene of interest”. Fold change was calculated as follows, DT+1/C-1 and C+64/C-1.

### CDKN2A, telomerase and anti-oxidant gene expression measurement

CDKN2A expression was measured using TaqMan Gene Expression Assay (cat # HS00923894_m1; Applied Biosystem) on a QuantStudio 5 qPCR instrument. The total qPCR reaction of 20 µl contained 5 µl cDNA, 10 µl TaqMan Multiplex Master Mix (cat # 4461882; Applied Biosystem), 1 µl GAPDH TaqMan Assay (cat # 4485712; Applied Biosystem), 1 µl CDKN2A GAPDH TaqMan Assay, (cat # 4331182; Applied Biosystem) and ddH_2_O. TaqMan GAPDH Assay (cat # 4485712; Applied Biosystem) was added to each run as an endogenous control.

Telomerase expression was measured using the human hTERT TaqMan Assay (cat # Hs00972650_m1; Applied Biosystem. The total qPCR reaction of 20 µl, contained 5 µl cDNA, 10 µl TaqMan Multiplex Master Mix, 1 µl TERT TaqMan Assay, 1 µl GAPDH TaqMan Assay, and ddH_2_O.

Relative gene expression was measured for five genes, including superoxide dismutases (SOD1 # Hs00533490_m1; and SOD2 # Hs00167309_m1), catalase (CAT # Hs00156308_m1), glutathione S-transferase Kappa (GSTK1 # Hs01114170_m1) and nitric oxide synthase (NOS3 # Hs01574665_m1). Each 20 μl qPCR reaction contained, 5 μl cDNA, 10 μl TaqMan Multiplex Master Mix (cat # 4461882; Applied Biosystem), 1 μl GAPDH Assay, 1 μl of either SOD1/SOD2/CAT/GASTK1or NOS3 Assay and ddH_2_O. Thermal profile included 95 °C for 20 s, followed by 45 thermal cycles (95 °C for 1 s and 60 °C for 20 s).

### Cytokine levels

Cytokine levels in citrate-plasma at three time-points C-1, DT+1 and C+64 were quantified using the LEGENDplex Multi-Analyte Flow Assay Kit (cat # 740118; BioLegend), a bead-based immunoassay referring to the basic principle of sandwich immunoassays, according to manufacturer´s instruction^34^. This kit was used to analyze 13 cytokines, including IL-1β, IFN-α2, IFN-γ, TNF-α, MCP-1 (CCL2), IL-6, IL-8 (CXCL8), IL-10, IL-12p70, IL-17A, IL-18, IL-23, and IL-33 simultaneously, as described in Reuling et al.^35^.

### Standard biochemical blood tests

Standard biochemical blood tests were performed to assess liver enzymes and renal function during the challenge, and included aspartate aminotransferase (AST), alanine aminotransferase (ALT), gamma-glutamyl transferase (γGT), alkaline phosphatase (ALP), lactate dehydrogenase (LD), and total bilirubin. All clinical laboratory data refer to standard tests performed on peripheral whole blood and reference laboratory values are found in Table S9, and Reuling et al.^35^. Liver function test abnormalities (#LFT) were assessed at three time-points (C-1, DT+1 and C+64), and were graded based on grade 1: 1.1.–2.5X ULN, grade 2: 2.6–5.0 × ULN, grade 3: > 5.0X ULN, using an adaptation of the World Health Organization (WHO) Adverse Event Grading System 2003, as described in Reuling et al.^26^.

### Statistical analysis

A cubic spline mixed model with restricted maximum likelihood was used to investigate telomere dynamics in association with host factors (age, sex, weight and treatment group), including individual ID as a random factor. These adjustments were made for each variable separately in a univariate model, and jointly for all variables in a multivariate model. Time was modeled using restricted randomized cubic splines, connected by knots and constricted to follow a linear distribution before the first and after the last knot^36^. Splines were randomized and fitted to three knots using the Stata statistic software, version 15 (StataCorp. 2017, Stata Statistical Software: Release 15. College Station, TX: StataCorp LLC). To investigate whether the level of parasite density was influenced by any host factor, we ran a separate cubic spline mixed model fitted by restricted maximum likelihood, including parasite density as outcome variable. Parasite density was log-transformed as values were not normally distributed. A paired t-test was used to investigate the change of TL, gene expression and cytokine levels on C-1, DT+1 and C+64. All variable values were log-transformed before paired t-test and for all further analysis. To investigate the potential correlation between variables, we used the mathematical and topological features of Spearman’s correlation (r_s_) and visualized it as arc diagram using R-studio, version 1.1.442, http://www.R-priject.org^37^.

Next, we performed a three-step analysis. First, we used Hierarchical Clustering to obtain a dendrogram that assembles variables based on similarities of variables according to time-points (C-1, DT+1 and C+64). We included the variables that showed significant change between C-1 and DT+1. Second, we used the Principal Component Analysis (PCA) approach to reduce the number of variables to avoid multiple co-linearities among the variables, using the two-clusters based on hierarchical cluster analysis. PCA is an unsupervised learning algorithm providing dimensions (linear correlations) along with the data that are separable, and reduces the noise associated with data whilst increasing its robustness. From the PCA analysis, we have selected the first three PCA axes (PC1, PC2, PC3 with Eigenvalues > 1), in total explaining 78.6% of the variance. Third, we did Factor analysis, based on our PCA results with three Factors, using Varimax method (JMP, Version 14.0). We used the individual Factor values on each of the three axes as dependent variable in a cubical spline mixed model to investigate how these values varied as a function time, age and sex. Time was fitted as cubical spline function, age and sex as fixed factors and individual ID as random factor.

## Results

### Telomere length dynamics during malaria infection

Sixteen healthy, malaria-naïve individuals were infected once with *P. falciparum* parasites by exposure to five infected *Anopheles* mosquitoes, and followed until day 64 post inoculation (PI). Blood samples were collected before infection and twice a day during day 6–15, daily during day 16–24, then every second day until day 45, and on day 64, respectively. *P. falciparum* parasites were detected by real time qPCR in peripheral blood from day 7–9 and reached a maximum level of 22,092 *Pf/*mL (median parasites/mL blood) between days 10–13 post challenge. After administration of the first treatment (DT+1) between days 9–13, very low levels of parasites were detected, while after the second treatment between days 20–23 all participants were qPCR negative (Fig. 1B). Compared to natural acute *Plasmodium* infection in human (< 0.1 to 21% iRBC)^1^, peak parasite density in study participants of the CHMI ranged from 0.000062 to 0.0049% iRBC (median 22.09 *Pf*/µL), indicating the low parasitic CHMI protocol. No serious adverse events were reported during the study period.

Telomere length (TL) was measured in peripheral blood (n = 481) from 29 to 31 samples per participant (Fig. 1, Table S2). Significant change in TL dynamics in peripheral blood was observed over the study period (Wald χ^2^ = 26.13, N = 139, p < 0.001, Fig. 1A, Table1). *P. falciparum* infection accelerated telomere shortening, with the shortest telomeres observed between day 10–13 post challenge (lme; p = 0.031, Fig. 1A) concurrent with maximum parasite count (197,500 *Pf/*mL blood; Fig. 1B,C). Restoration of TL was observed already upon day 22 and was fully established upon day 64 post infection (p > 0.05, Fig. 1A). There was no significant effect of age, weight and type of anti-malarial treatment, however, females had longer telomeres than males (Table 1, Figure S1A). This sex difference remained significant prior, during and post infection (two-sample t-test, all p < 0.001). Overall mean TL for females was 13.79 kb (SD 3.67, 95% CI 13.41–14.17) compared to males 10.88 kb (SD 2.84, 95% CI 10.37–11.39, two-sample t-test t_1, 261_ = 9.01, p < 0.001). Telomere length and parasite density were negatively correlated (lme; p < 0.006, Fig. 1C) after adjusting for age, sex and type of antimalarial treatment (Table S3). Furthermore, parasite density was not affected by any additional factor in our mixed model (Table S4, Figure S1B).

**Table 1:** Association between host factors and telomere dynamics during the controlled human malaria infection (n = 16,481 observations).

Parameters	Univariate analysis				Multivariate analysis			
	Coef.	SE	Z	p-value	Coef.	SE	Z	p-value
Time	0.023	0.008	2.71	0.007				
Time spline1					− 0.053	0.024	− 2.16	0.031
Time spline 2					0.117	0.035	3.30	0.001
Age (years)	− 0.255	0.236	− 1.08	0.281	− 0.369	0.224	− 1.65	0.099
Female	2.919	1.388	2.10	0.035	2.889	1.394	2.07	0.038
Weight (kg)	− 0.064	0.067	− 0.95	0.343	− 0.05	0.074	− 0.68	0.499
Treatment	− 0.461	0.604	− 0.76	0.446	− 0.601	0.624	− 0.96	0.335

In summary, low-density *P. falciparum* infection accelerated telomere shortening in all study participants within 64 days. Complete restoration of TL followed from day 22 and was fully established upon day 64 post infection.

### Kinetics of cellular aging, cytokine expression and anti-oxidant levels prior, during and post infection

**Figure 2:**
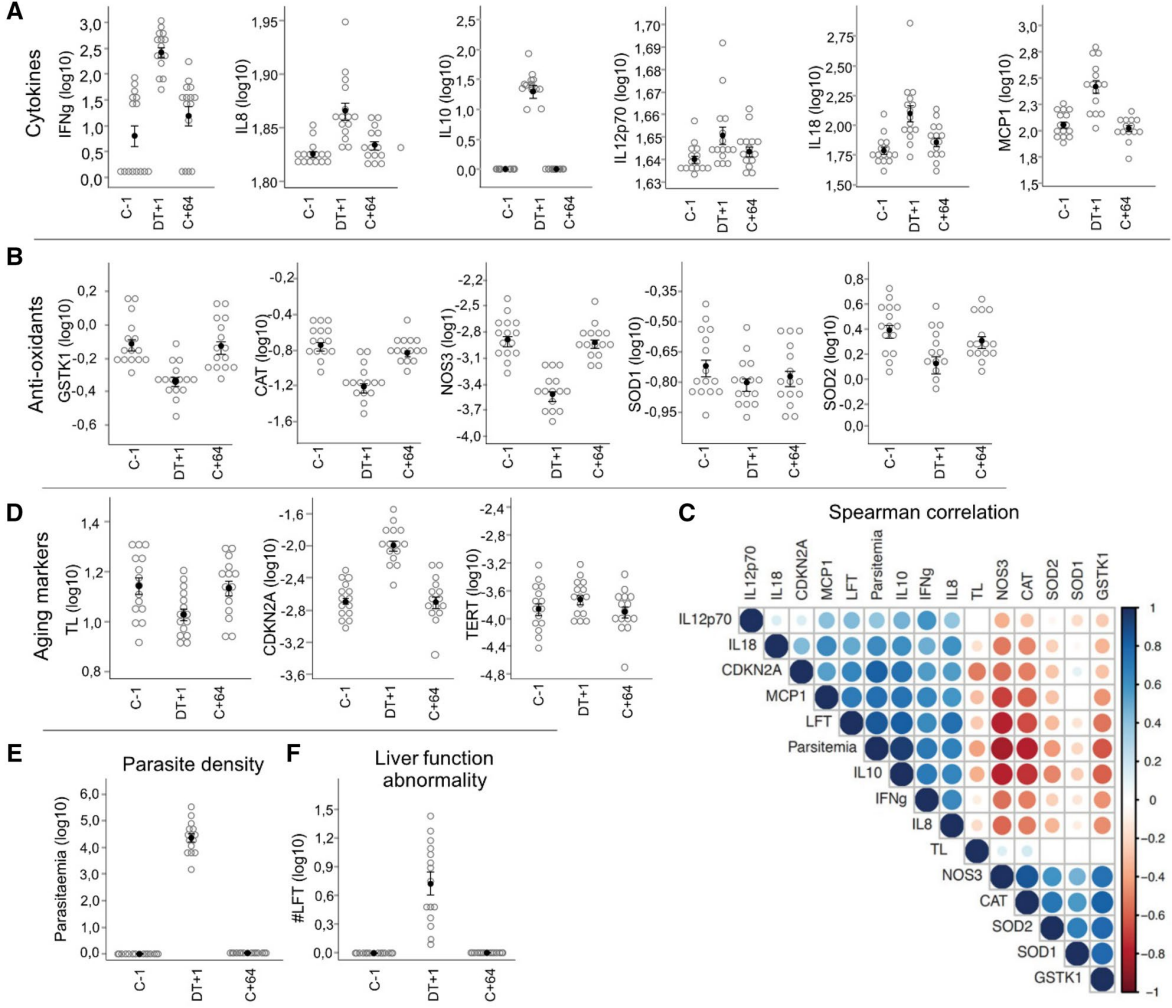
Changes in cellular aging markers, parasite density, liver function abnormalities, cytokine levels and gene expression before, during and after a controlled human malaria infection. Log Δ*C*_*T*_ values were used for graphical presentation of parasite levels and gene expression in all study participants (n = 16) prior (C-1), during (DT+1/day after treatment) and post infection (C+64). (**A**) log changes of cytokines, including (from left to right) IFN-γ, IL-8, IL-10, IL-12p20, IL-18, and MCP1. Mean values for the absolute peak parameters of cytokines in plasma at DT+1: IFNγ 179.3 pg/mL; IL-8 72 pg/mL; IL-10 22.9 pg/mL; IL-12p70 44.1 pg/mL; IL-18 108.1 pg/mL; MPC1 263.3 pg/mL. (**B**) log changes of anti-oxidants in 15/16 study participants, including (from left to right) GSTK1, CAT, NOS3, SOD1, and SOD2. Open circles represent individual data time points, filled circles denote for mean ± SE. (**C**) Arc diagram showing inter-correlation (Spearman’s correlation) of study variables with a significance level of p < 0.05. Circle size indicates the strength of correlation, and color of the scale bar denotes the nature of correlation (dark blue (1) corresponding to positive correlation, and dark red (− 1) corresponding to negative correlation (Table S7). For data visualization we used R-studio, version 1.1.442; https://www.R-project.org^37^. (**D**) log changes of aging markers, including (from left to right) telomere length, CDKN2A expression, and TERT expression. (**E**) Log change of *P. falciparum* levels and (**F**) liver function abnormalities, based on biochemical functional assays at three study time-points. TL = telomere length, #LFT = liver function test abnormality score, CDKN2A = cyclin dependent kinase inhibitor 2A, TERT = telomerase.

Results reveal the manifestation of a pro-inflammatory status, with the increase of six cytokines (all p < 0.05, Fig. 2A, Table S6) upon day after treatment 1 (DT+1), followed by a decrease in their expression post final treatment (C+64, all p < 0.05), with exception of IL-12p70. When comparing pre infection (C-1) with post infection and final treatment (C+64). IL-10 and MCP-1 levels decreased (all p > 0.05), however, IFNγ, IL-8 and IL-18 remained marginally high (Fig. 2A, Table S6). INFα and IL-17A levels were gradually increased and significantly higher on C+64 compared to C-1 (all p < 0.05 Table S6). IL-1β, TNF-α, IL-6, and IL-23 were not detected during the entire study (Table S1). Inflammatory cytokine expression was positively correlated with parasite density, CDKN2A levels, liver function test abnormalities score (#LFT), and negatively correlated with TL (Spearman’s correlation, all p < 0.05, Fig. 2C, Table S7).

Concurrent with the pro-inflammatory status, we found a significant decrease in anti-oxidant gene expression upon DT+1 compared to C-1 (all p < 0.05, Fig. 2B, Table S8), indicating disrupted oxidant-antioxidant balance during infection. Anti-oxidant levels restored upon C+64 (difference C-1 vs C+64 all p > 0.05), with exception of SOD1 levels, which marginally increased. In addition, anti-oxidant gene expression was negatively correlated with parasite density, inflammatory cytokines, #LFT and CDKN2A levels, while positively correlated with TL (Spearman’s correlation, all p < 0.05, Fig. 2C, Table S7). These results suggest malaria parasitaemia triggered an oxidative stress response that negatively affects telomere shortening, and implements cellular senescence.

Kinetics of cellular aging markers changed significantly between the day prior to infection (C-1) and day post treatment 1 (DT+1), with a significant decrease in TL (p = 0.004, Fig. 2D) and elevated CDKN2A expression levels upon DT+1 (p < 0.001, Fig. 2D); both markers were significantly correlated with parasite density (all p < 0.05, Fig. 2C). Upon our final time point (C+64), TL and CDKN2A expression reversed to base line values, and showed no significant difference in markers between C-1 and C+64 (all p > 0.05, Fig. 2D). No significant change in telomerase expression (TERT) was observed throughout the study period of 64 days. Differences in cellular aging markers between time points are shown in Table S5. Liver function abnormalities (#LFT) peaked shortly after treatment and returned to baseline values before the end of the study, similar to parasite density (Fig. 2E,F)^26^. The most prevalent #LFT during the CHMI were ALT/AST elevations (n = 16/16)^26^. As described in Reuling et al., this study noted one grade three abnormality (elevated levels of ALT, n = 8; elevated levels of AST, n = 7). All study participants showed mild to severe ALT/AST elevations; 5/16 (31%) mild (grade 1); 3/16 (19%) moderate (grade 2), and 8/16 (50%) participants showed severe (grade 3) (up to 25 × ULN) ALT/AST elevations^26^. Besides the positive correlation of #LFT with inflammatory parameters, as reported in Reuling et al., #LFT were further positively correlated with parasite density and CDKN2A levels, while negatively correlated with TL (Spearman’s correlation, all p < 0.05, Fig. 2C, Table S7).

In summary, coinciding with relatively low parasite density, we see a pro-inflammatory profile, decreased anti-oxidant gene expression, elevated CDKN2A levels and accelerated telomere shortening at 10–13 days post challenge, suggesting parasite-mediated mechanisms affecting cellular aging markers.

### Expression profile of study markers during the CHMI

#### Hierarchical cluster and principal component analysis

Using a Hierarchical Clustering Analysis we show two distinct clusters for the measured markers by separating DT+1 from time point C-1 and C+64, underlining the similar expression profile at the time point prior (C-1) and post infection (C+64), and highlighting the distinctive difference to the marker profile during infection (DT+1). This difference is characterized by an increase in cytokine levels and CDKN2A expression, higher #LFT, lower anti-oxidant gene expression and reduced TL at DT+1 (Fig. 3A), compared to C+64 and C-1.

**﻿Figure 3:**
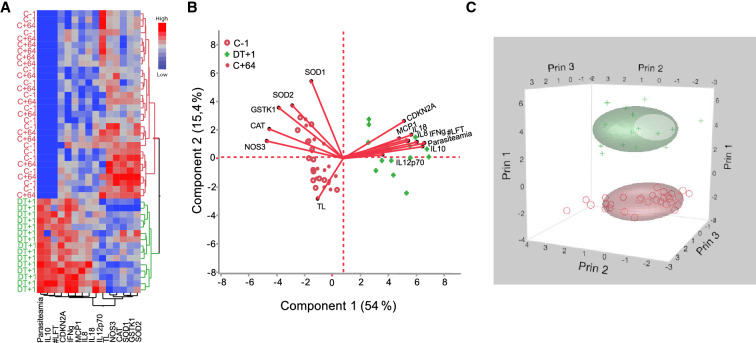
Heatmap and Principal Component Analysis (PCA) showing clusters of cellular aging markers (telomere length, telomerase activity and CDKN2A expression), inflammatory cytokines and anti-oxidant gene expression. (**A**) Heat-map displaying results of our hierarchical cluster analysis and differential expression level of grouped study variables at time points C-1, DT+1 and C+64. TL = telomere length, #LFT = liver function test abnormality score, CDKN2A = cyclin dependent kinase inhibitor 2A. (**B**) 2D PCA, separating cytokines, anti-oxidants, and TL during time points C-1 (red circles), DT+1 (green plus symbol), and C+64 (red dots). (**C**) K-mean cluster separating variables into three PC axes (Prin1-3) to visualize the separation between time point C-1 and C+64 (red circle) from DT+1 (green plus-symbol). Data visualization is based on analysis in Stata software (StataCorp. 2017, Stata Statistical Software: Release 15. College Station, TX: StataCorp LLCVersion 15).

To reduce the co-linearity among response variables, we performed an unsupervised PCA analysis that produced three main principal component axes with Eigenvalue > 1, explaining collectively 78.5% of the variance in the response (Fig. 3B,C, Table S10). PC 1 explains 54% variance (χ^2^ = 690.09, df = 103.89, p < 0.001), PC 2 explains 15.4% variance (χ^2^ =  377.33, df = 103.522, p < 0.001) and PC 3 explains 9.1% variance (χ^2^ = 264.56, df = 92.75, p < 0.001, Table S10), followed by a long tail of low explanatory axes.

#### Factor analysis explaining variance within components

To explain the variance within our PCA results, we conducted Factors analysis, which resulted in three main significant factors explaining in total 78.6% of variance (Factor1 = 42.8, Factor 2 = 24.8 and Factor 3 = 11.0, Table S11). Factor 1 mainly consisted of CDKN2A and cytokines, Factor 2 consisted of anti-oxidants, and Factor 3 consisted of TL (Table S11). We performed a cubical spline mixed model to investigate the effect of infection, age and sex using Factors as our dependent variable. Factors 1, 2 and 3 were significantly affected by malaria infection (all p < 0.001), however, there was no effect of sex and age (all p > 0.05, Table 2).

**Table 2:** Association between PCA Factors and host factors during the controlled human malaria infection (n = 16,139 observations).

	Variables	Coef.	SE	Z	p-value
Factor 1	Time_Spline1	0.157	0.015	10.01	< 0.001
	Time_Spline 2	− 0.426	0.039	− 10.68	< 0.001
	Female (yes)	0.125	0.224	0.56	0.577
	Age (years)	0.048	0.040	1.19	0.234
Factor 2	Time_Spline1	− 0.073	0.018	− 3.39	< 0.001
	Time_Spline 2	0.191	0.047	4.04	< 0.001
	Female (yes)	− 0.708	0.406	− 1.74	0.081
	Age (years)	0.142	0.073	1.93	0.053
Factor 3	Time_Spline1	− 0.048	0.020	− 2.34	0.020
	Time_Spline 2	0.139	0.052	2.68	0.007
	Female (yes)	0.624	0.462	1.35	0.177
	Age (years)	− 0.068	0.084	− 0.81	0.418

## Discussion

Findings from this study reveal the interconnectivity of aging markers (TL and CDKN2A) and aging-related processes (inflammation and oxidative stress) during a single malaria infection. Existing studies in malaria are often restricted in individual heterogeneity, history of malaria exposure and the effect of environmental factors, such as co-infections; key factors that are well-known to influence TL and other cellular aging processes^1,4^. This CHMI model shows that extremely low parasite exposure causes inflammation and oxidative stress that lead to accelerate telomere shortening and cellular senescence. However, the parasite-mediated effect was transient, similar to results in travelers with fulminant clinical *P. falciparum* infection, where the effect on cellular aging was most pronounced 1 month after treatment, and almost reversed aging dynamics 12 months post hospital admission and in the absence of reinfection^1^. In contrast to the traveler study, parasite levels in this study were extremely low and strictly controlled by low doses of subcurative treatment, which prevented clinical manifestations in all participants and provided an extended infection period. In addition, the current study specifically characterizes the relationship among TL, CDKN2A, inflammation, oxidative stress and tissue injury, prior, during and post low parasitic infection. Furthermore, parasite density and TL measured in daily samples add detailed information on the link between parasite density and telomere dynamics, revealing the exact time of telomere reduction due to infection, and the time point of TL restoration. These data provide novel information to the interdisciplinary field of aging and infectious disease research, which has not been shown in previous studies.

Both, cells of the innate and adaptive immune response produce free radicals, reactive oxygen species (ROS) and inflammatory factors for the defense and destruction of pathogens. Inflammation and oxidative stress are interlinked processes, which play an important role in invoking an immune response, but also contribute to age-related loss of homeostasis and health^24^. The overproduction of pro-oxygen compounds can trigger activation of leukocytes, enhancing the expression of cytokines, chemokines and other inflammatory mediators^38^. An imbalance between oxidants and anti-oxidants, and a lack of anti-inflammatory defense systems has been shown to cause cellular damage and aging^39^. In terms of cellular aging, increase in pro-inflammatory cytokines has been shown to induce a reinforced senescence status, impacting cell plasticity and stemness features^40^. In this study, secretion of several inflammatory cytokines was elevated, while cytokine levels of IL-1β and IL-6, key players in clinical malaria^41^, together with TNFα were not detected. The absence of IL-1β and IL-6 and TNFα during CHMI is not unusual as reported in other controlled malaria studies^42,43^. This might be due to strict monitoring and early drug treatment of parasitaemia, compared to high parasite levels and severe clinical pathology in natural malaria infection. Another explanation could be the use of citrate plasma for cytokine measurement, which is known to compromise the IL-6 detection^44^.

Independent from the immunologic stimuli caused by acute malaria, which involves increased pro-inflammatory levels of TNF-α, IL-1β, IL-12 and IL-18^20,21^, inflammation induced by the malaria parasite and the host’s production of ROS can give rise to pronounced DNA damage^45^, with telomeres being preferential targets for oxidative damage-leading to a senescence- associated inflammatory response, including elevated IL-6, IL-8 and INF-γ levels^46^. Critically short telomeres or damage in these non-coding sequences are known mechanisms inducing cellular senescence^47^. Accumulation of senescent cells in turn, leads to the acquisition of the senescence-associated secretory phenotype (SASP), which has a deleterious effect on the tissue microenvironment by further increasing pro-inflammatory cells, most prominently inducing IL-6^48,49^. The pro-inflammatory phenotype consequently leads to an imbalance of the cellular redox state, generating even higher amounts of oxidative stress. It is well established that deprivation of anti-oxidant mechanisms along with oxidative damage play a major role in several pathways leading to cellular senescence^50^, mainly reasoned by declining levels of IL-10 that cause inflamm-aging (an aging-associated inflammation state^45,51^.

Results from this study show that anti-oxidant gene expression was down regulated for SOD1, SOD2, NOS3, CAT and GSTK during infection, which is in line with results published by Reuling et al.^35^. Imbalanced formation of oxidizing species has been a relatively frequent finding in severe malaria infection^52,53^. Oxidative stress is commonly observed during pathogenesis as a consequence of (a) inflammatory processes initiated by the host’s response to infection^52,53^, (b) reactive species produced directly by the parasite^52^, (c) *Plasmodium* infected erythrocytes producing twice as much hydroxyl radicals compared to uninfected erythrocytes^54^ and (d) anti-malaria treatment^55–57^.

In this study, all markers (senescence, parasitaemia, cytokines, and oxidative stress) were assessed by extracting RNA from whole blood. In previous studies, using an exploratory approach with mass cytometry, followed by targeted flow cytometry in individuals with naturally acquired malaria, we have shown an expansion of T helper cells and memory B cells^58^, suggesting that these might be abundant cell type changes. However, we did not find that change in cell composition (of monocytes, neutrophils and lymphocytes) was associated with telomere length in our previous study of naturally acquired malaria in travelers^1^.

Recently, Reuling et al.^35^ reported an association between parasitic density, inflammation parameters and liver injury (including data from this study) during an uncomplicated malaria infection. Here, we further explored the relationship between liver injury and cellular senescent markers (telomere length and CDKN2A). The association of liver function test abnormality score with cytokines, anti-oxidant enzymes and CDKN2A expression in this study suggests that increased cytokine production and low anti-oxidant gene expression in whole blood might also reflect DNA damage (including telomere shortening and elevated CDKN2A expression) in human hepatic cells^59,60^. These findings are concurrent with an experimental malaria study in birds showing that malaria infection causes systemic stress that shortens TL in multiple body tissues, including blood, liver, lung, kidney, spleen, brain and heart^6^.

Despite complete restoration of TL post CHMI, we found no significant change in TERT expression during the study period. However, expression was slightly higher upon day after treatment 1 (DT+1), which might be enough to restore TL (Fig. 1A). A moderate suppression and activation of TERT over time was also observed in our previous study in travelers^1^. An adjacent hypothesis of telomere restoration and increased telomerase activity is the activation and differentiation of T lymphocytes^61^, as part of humoral immune response. Majority of activated cells die during immune activation or drug treatment, however, memory T and B cells with longer telomere length remain in circulation for longer time, which could partially explain telomere restoration in whole blood (Fig. 1). Another plausible explanation could be the fact that infection leads to an increased cell turn-over upon activated immune response or drug treatment, in which case our measured TL would reflect DNA dynamics of recently divided cells with shorter telomere. Another possible explanation for accelerated telomere shortening and increased CDKN2A levels in peripheral blood could be a change in cell composition during infection. However, a study by Asghar et al.^20^ showed that TL was not linked to change in proportion of monocytes, neutrophils and lymphocytes in blood over a year follow up. Nevertheless, further studies are needed to pinpoint the exact mechanisms behind telomere restoration in blood.

In conclusion, using a novel approach of controlled human malaria infection with daily samples, we characterized the impact of low-density malaria infection on cellular aging markers, and their complete restoration after successful treatment. Our work presents an integrative model of hallmarks of aging during infection, combining data of aging marker dynamics with inflammation and oxidative stress, generated by extremely low levels of malaria parasitaemia. Our findings stress that even short term exposure to human pathogens generates a high host response, given the fact that even low parasite exposure impacts cellular aging. These results further urge the need to explore the impact of malaria in endemic areas, considering that continuous exposure to the parasite, repeated clinical episodes, and chronic asymptomatic infections could potentially lead to permanent long-term consequences on cellular aging and immune function, which may cause irreversible health consequences in human. We therefore stress future studies to evaluate infection-induced long term consequences in human living under chronic disease exposure, to not only emphasize the need of malaria eradication, but also imply intervention methods based on the parasite-altered cellular mechanisms in the host.

## Supplementary Information

Supplementary Information.

## Data Availability

All data are available upon request to Muhammad Asghar (asghar.muhammad@ki.se).
