# Supplementary material for: Biomarkers of cellular aging during a controlled human malaria infection
Source: Sci Rep. 2021 Sep 21;11:18733. doi: 10.1038/s41598-021-97985-y (PMC8455531; doi:10.1038/s41598-021-97985-y)
Supplement: Supplementary file 1 — Supplementary Information. [file 41598_2021_97985_MOESM1_ESM.docx]

**Biomarkers of cellular aging during a controlled human malaria infection**

Aurelie Miglar^1,2,3^, Isaie J. Reuling^4^, Xi Zen Yap^4^, Anna Färnert^1,2,3^, Robert W. Sauerwein^4,5^, Muhammad Asghar^1,2,3^*

*^1^Division of Infectious Diseases, Department of Medicine Solna, Karolinska Institutet, Sweden. ^2^Department of Infectious Diseases, Karolinska University Hospital, Sweden.*

*^3^Center for Molecular Medicine, Karolinska Institutet, Sweden.*

*^4^Department of Medical Microbiology, Radboud University Medical Center, Geert Grooteplein 28, Microbiology 268, 6500 HB, Nijmegen, The Netherlands.*

*^5^TropIQ Health Sciences, Transistorweg 5-C02, 6534 AT, Nijmegen, The Netherlands*

^*^**Correspondence:** Muhammad Asghar

E-mail: [Asghar.muhammad@ki.se](mailto:Asghar.muhammad@ki.se); Telephone: +46-08 524 825 04

Running Title: Effect of malaria on cellular aging in human

Key Words: *CHMI, Malaria, Oxidative stress, Inflammation, Telomeres, CDKN2A, Aging*

**Supplementary files**

**Figure S1. Telomere length (kb) and parasite density (log) plotted by sex.**

(A) Telomere length in females (red line) was longer than in males (blue line, Coef.= -3.51, SE=1.5, Z=2.34, p=0.019. Overall mean TL for females was 13.79 kb (SD 3.67, 95% CI 13.41-14.17) compared to males 10.88 kb (SD 2.84, 95% CI 10.37-11.39, two-sample t-test t1, 261 = 9.01, p < 0.001). (B) Parasite density peaked between day 10-13, with no significant difference between sexes (Coef.= 0.331, SE=0.234, Z=-1.41, p=0.158). Mean parasite density for females was 47635 *Pf*/mL, and for males 8808 *Pf*/mL. Solid blue and red lines represent predicted means, with red and blue dots representing the mean value ± standard error at each time point.

**Table S1.** **Paired t-test significance levels (p-values) of mean difference of cellular aging markers, cytokine levels and anti-oxidant genes prior (C-1), during (DT+1) and post infection (C+64).**

|  |  | **C-1 /DT+1** | **C-1 / C+64** | **DT+1 /C+64** |
| --- | --- | --- | --- | --- |
| a | **TL** | **0.002** | 0.343 | **0.004** |
|  | **CDKN2A** | **0.001** | 0.98 | **0.001** |
|  | **TERT** | 0.135 | 0.96 | 0.072 |
| b | **IL-1β** |  |  |  |
|  | **IFN-α2** | 0.95 | **0.005** | **0.008** |
|  | **IFN-γ** | **0.001** | **0.008** | **0.001** |
|  | **TNF-α** |  |  |  |
|  | **MCP-1** | **0.001** | 0.292 | **0.001** |
|  | **IL-6** |  |  |  |
|  | **IL-8** | **0.001** | **0.030** | **0.005** |
|  | **IL-10** | **0.001** |  | **0.001** |
|  | **IL-12p70** | 0.023 | 0.057 | 0.141 |
|  | **IL-17A** | 0.057 | **0.031** | **0.008** |
|  | **IL-18** | **0.001** | **0.017** | **0.001** |
|  | **IL-23** |  |  |  |
|  | **IL-33** | 0.090 | 0.201 | **0.041** |
| c | **SOD1** | **0.017** | 0.077 | 0.435 |
|  | **SOD2** | **0.001** | 0.055 | **0.027** |
|  | **GSTK** | **0.001** | 0.315 | **0.001** |
|  | **NOS3** | **0.001** | 0.584 | **0.001** |
|  | **CAT** | **0.001** | 0.088 | **0.001** |

Significance levels (p-values, paired t test) of mean differences during the study time points C-1, DT+1 and C+64 of a) cellular aging markers, b) cytokines and c) anti-oxidant markers. Green shade indicates high significance (p < 0.01); light green shade indicates borderline significance, grey shade indicates a non-significance (p > 0.05).

**Table S2.** **Study overview: total number of measurements and analyzed markers per study individual (n= 16).**

| **ID (n=16)** | **D.F. 1960** | **D.L. 1970** | **E.A. 2257** | **D.S.**  **2878** | **E.K. 2921** | **D.N. 3146** | **D.B. 3259** | **D.O. 3270** | **D.V. 3366** | **E.B. 3368** | **D.K. 3382** | **E.D. 3899** | **D.X. 3903** | **D.Y. 3911** | **E.N. 3934** | **E.F. 3963** | **N_individuals_** | **N_samples_** | |  |
| --- | --- | --- | --- | --- | --- | --- | --- | --- | --- | --- | --- | --- | --- | --- | --- | --- | --- | --- | --- | --- |
| **N_time-points_** | 30 | 30 | 30 | 31 | 30 | 30 | 31 | 31 | 31 | 31 | 30 | 31 | 30 | 31 | 30 | 29 |  | |  | |
| **TL** | 30 | 30 | 30 | 31 | 30 | 30 | 30 | 31 | 31 | 30 | 29 | 30 | 29 | 31 | 30 | 29 | 16 | | 481 | |
| **IL-1β** | 3 | 3 | 3 | 3 | 3 | 3 | 3 | 3 | 3 | 3 | 2 | 3 | 3 | 3 | 3 | 3 | 16 | | 47 | |
| **IFNα2** | 3 | 3 | 3 | 3 | 3 | 3 | 3 | 3 | 3 | 3 | 2 | 3 | 3 | 3 | 3 | 3 | 16 | | 47 | |
| **IFNγ** | 3 | 3 | 3 | 3 | 3 | 3 | 3 | 3 | 3 | 3 | 2 | 3 | 3 | 3 | 3 | 3 | 16 | | 47 | |
| **TNFα** | 3 | 3 | 3 | 3 | 3 | 3 | 3 | 3 | 3 | 3 | 2 | 3 | 3 | 3 | 3 | 3 | 16 | | 47 | |
| **MCP-1** | 3 | 3 | 3 | 3 | 3 | 3 | 3 | 3 | 3 | 3 | 2 | 3 | 3 | 3 | 3 | 3 | 16 | | 47 | |
| **IL-6** | 3 | 3 | 3 | 3 | 3 | 3 | 3 | 3 | 3 | 3 | 2 | 3 | 3 | 3 | 3 | 3 | 16 | | 47 | |
| **IL-8** | 3 | 3 | 3 | 3 | 3 | 3 | 3 | 3 | 3 | 3 | 2 | 3 | 3 | 3 | 3 | 3 | 16 | | 47 | |
| **IL-10** | 3 | 3 | 3 | 3 | 3 | 3 | 3 | 3 | 3 | 3 | 2 | 3 | 3 | 3 | 3 | 3 | 16 | | 47 | |
| **IL-12p70** | 3 | 3 | 3 | 3 | 3 | 3 | 3 | 3 | 3 | 3 | 2 | 3 | 3 | 3 | 3 | 3 | 16 | | 47 | |
| **IL-17A** | 3 | 3 | 3 | 3 | 3 | 3 | 3 | 3 | 3 | 3 | 2 | 3 | 3 | 3 | 3 | 3 | 16 | | 47 | |
| **IL-18** | 3 | 3 | 3 | 3 | 3 | 3 | 3 | 3 | 3 | 3 | 2 | 3 | 3 | 3 | 3 | 3 | 16 | | 47 | |
| **IL-23** | 3 | 3 | 3 | 3 | 3 | 3 | 3 | 3 | 3 | 3 | 2 | 3 | 3 | 3 | 3 | 3 | 16 | | 47 | |
| **IL-33** | 3 | 3 | 3 | 3 | 3 | 3 | 3 | 3 | 3 | 3 | 2 | 3 | 3 | 3 | 3 | 3 | 16 | | 47 | |
| **CDKN2A** | 3 | 3 | 3 | 3 | 3 | 3 | 3 | - | 3 | 3 | 2 | 3 | 3 | 3 | 3 | 3 | 15 | | 44 | |
| **TERT** | 3 | 3 | 3 | 3 | 3 | 3 | 3 | - | 3 | 3 | 2 | 3 | 3 | 3 | 3 | 3 | 15 | | 44 | |
| **SOD1** | 3 | 3 | 3 | 3 | 3 | 3 | 3 | - | 3 | 3 | 2 | 3 | 3 | 3 | 3 | 3 | 15 | | 44 | |
| **SOD2** | 3 | 3 | 3 | 3 | 3 | 3 | 3 | - | 3 | 3 | 2 | 3 | 3 | 3 | 3 | 3 | 15 | | 44 | |
| **GSTK** | 3 | 3 | 3 | 3 | 3 | 3 | 3 | - | 3 | 3 | 2 | 3 | 3 | 3 | 3 | 3 | 15 | | 44 | |
| **NOS3** | 3 | 3 | 3 | 3 | 3 | 3 | 3 | - | 3 | 3 | 2 | 3 | 3 | 3 | 3 | 3 | 15 | | 44 | |
| **CAT** | 3 | 3 | 3 | 3 | 3 | 3 | 3 | - | 3 | 3 | 2 | 3 | 3 | 3 | 3 | 3 | 15 | | 44 | |

In total 16 healthy volunteers were enrolled in the Controlled human malaria infection study. Study individuals account for 29-31 time points each‧ for which continuous data on telomere dynamic is available (N= 481). Cellular markers were measured on three time points for each individual respectively, with exclusion of one individual lacking one time points (C+64). One individual was dropped for gene expression analysis‧ due to errors in cDNA synthesis. Total number of analyzed markers N= 919.

**Table S3**. **Correlation of telomere length and parasite density (n = 16, observations = 139).**

| **Parameters** | **Coef.** | **SE** | **Z** | **p-value** |
| --- | --- | --- | --- | --- |
| **Age (Years)** | -0.248 | 0.238 | -1.04 | 0.297 |
| **Female (Yes)** | 3.512 | 1.503 | 2.34 | 0.019 |
| **Treatment** | -0.355 | 0.596 | -0.6 | 0.551 |
| **Parasite density(Pf/mL)** | -0.545 | 0.197 | -2.77 | 0.006 |

**Table S4. Association between host factors and parasite density after malaria infection (n = 16, observation = 139).**

| **Parameters** | **Coef.** | **SE** | **Z** | **p-value** |
| --- | --- | --- | --- | --- |
| **Female (Yes)** | 0.331 | 0.234 | 1.41 | 0.158 |
| **Age (Years)** | -0.027 | 0.042 | -0.06 | 0.949 |
| **Weight (kg)** | 0.01 | 0.013 | 0.76 | 0.446 |
| **Treatment** | 0.021 | 0.107 | 0.19 | 0.846 |

**Table S5. Mean difference of aging markers during the controlled human malaria infection.**

**Paired t-test significance level p= 0.05. 95% confidence interval (95% CI)”.**

|  | **C-1 /DT+1** | **C-1 / C+64** | **DT+1 /C+64** |
| --- | --- | --- | --- |
| **TL** | Mean diff.= 0.12  95%CI = -0.05 - -0.19  p= 0.002 | Mean diff.= 0.027  95%CI = -0.032 - 0.086  p= 0.343 | Mean diff.= -0.093  95%CI = -0.151 - -0.035  p= 0.004 |
| **CDKN2A** | Mean diff.= -0.699  95%CI = -0.785 - -0.614  p< 0.001 | Mean diff.= -0.002  95%CI = -0.136 - 0.133  p= 0.98 | Mean diff.= -0.698  95%CI = 0.577 - 0.818  p< 0.001 |
| **TERT** | Mean diff.= -0.153  95%CI = -0.36 - 0.054  p= 0.135 | Mean diff.= -0.005  95%CI = -0.204 - 0.195  p= 0.96 | Mean diff.= 0.15  95%CI = -0.015 - 0.316  p= 0.072 |

**Table S6. Mean difference of cytokine levels that significantly changed during the Controlled human malaria infection. Paired t-test significance level p= 0.05. 95% confidence interval of mean difference is presented as “95% CI”. Undetected cytokine levels (IL-1β, IL-6, IL-23 and TNFα) were excluded from the table.**

|  | **C-1 /DT+1** | **C-1 / C+64** | **DT+1 /C+64** |
| --- | --- | --- | --- |
| **IL-8** | Mean diff.= -0.0386  95%CI = -0.056 - -0.0208  p< 0.001 | Mean diff.= -0.010  95%CI = -0.019 - -0.001  p= 0.030 | Mean diff.= 0.028  95%CI = 0.01 - 0.047  p= 0.005 |
| **IL-10** | Mean diff.= -1.294  95%CI -1.526 - -1.062 p< 0.001 |  | Mean diff.= 1.294  95% CI= 1.062 - 1.526  p< 0.001 |
| **IL-17A** | Mean diff.= 0.002  95%CI= -0.001 - 0.004  p= 0.057 | Mean diff.= -0.009 95%CI= -0.0178 - -0.001 p= 0.031 | Mean diff.= -0.012  95%CI= -0.02 - -0.004  p= 0.008 |
| **IL-12p70** | Mean diff.= - 0.01  95%CI= -0.018 - -0.0016  p= 0.023 | Mean diff.= - 0.004 95%CI= -0.008 - 0.001  p= 0.057 | Mean diff.= 0.006 95%CI= -0.002 - 0.014  p= 0.141 |
| **IL-18** | Mean diff.= -0.302  95%CI= -0.403 - -0.201  p< 0.001 | Mean diff.= -0.076  95%CI= -0.137 - -0.0157  p= 0.017 | Mean diff.= 0.226  95%CI= 0.129 - 0.323  p< 0.001 |
| **IL-33** | Mean diff.= 0.006  95%CI= -0.001 - 0.001 p= 0.090 | Mean diff.= -0.012 95%CI= -0.032 - 0.007 p= 0.201 | Mean diff.= -0.018  95%CI= -0 .035 - -0.001  p= 0.041 |
| **IFNα** | Mean diff.= 0.001 95%CI= -0.019 - 0.02 p= 0.95 | Mean diff.= -0.077  95%CI= -0.0126 - -0.028  p= 0.005 | Mean diff.= -0.078  95%CI= -0.132- -0.023  p= 0.008 |
| **IFNγ** | Mean diff.= -1.627  95%CI= -2.081- -1.172 p< 0.001 | Mean diff.= -0.445  95%CI= -0.758 - -0.132  p= 0.008 | Mean diff.= 1.182  95%CI= 0.786 - 1.577  p< 0.001 |
| **MCP-1** | Mean diff.= -0.342  95%CI= -0.462 - -0.222 p< 0.001 | Mean diff.= 0.023 95%CI= -0.022 - 0.068 p= 0.292 | Mean diff.=0.365  95%CI= 0.247 - 0.483  p< 0.001 |

**Table S7. Spearmen correlation Matrix of response variables showing significant correlations (p <0.05).**

|  | **#LFT** | **TL** | **INFγ** | **IL-8** | **IL-10** | **IL12p70** | **IL-18** | **MCP1** | **NOS3** | **SOD1** | **SOD2** | **CAT** | **GSTK1** | **CDKN2A** |
| --- | --- | --- | --- | --- | --- | --- | --- | --- | --- | --- | --- | --- | --- | --- |
| **Parasite density** | 1.9E-12 | 7.4E-03 | 5.3E-09 | 3.7E-09 | 9.5E-17 | 7.6E-04 | 2.3E-07 | 1.7E-10 | 5.1E-11 | 2.1E-03 | 8.2E-07 | 1.8E-10 | 2.3E-07 | 1.3E-08 |
| **#LFT** | 0.0E+00 | 1.5E-02 | 9.6E-09 | 3.0E-10 | 3.8E-12 | 3.8E-04 | 1.1E-06 | 1.4E-09 | 2.3E-11 | 1.5E-03 | 1.5E-06 | 5.4E-10 | 1.7E-07 | 2.6E-07 |
| **TL** |  | 0.0E+00 | 4.0E-02 | 2.2E-02 | 1.1E-02 |  | 1.9E-02 | 1.0E-02 | 4.6E-02 |  |  | 4.8E-02 |  | 3.1E-04 |
| **INFγ** |  |  | 0.0E+00 | 1.8E-08 | 2.7E-09 | 3.0E-05 | 6.1E-06 | 2.0E-08 | 5.9E-09 | 6.0E-04 | 2.1E-06 | 1.1E-08 | 1.1E-07 | 9.0E-06 |
| **IL-8** |  |  |  | 0.0E+00 | 3.7E-09 | 6.0E-04 | 2.6E-07 | 3.2E-08 | 5.6E-09 | 1.4E-03 | 9.5E-07 | 1.1E-08 | 2.2E-07 | 2.5E-06 |
| **IL-10** |  |  |  |  | 0.0E+00 | 4.0E-04 | 3.3E-07 | 2.8E-10 | 1.3E-11 | 1.1E-03 | 2.3E-07 | 2.9E-11 | 6.1E-08 | 8.5E-08 |
| **IL12p70** |  |  |  |  |  | 0.00E+00 | 3.15E-01 | 1.04E-02 | 1.93E-03 | 3.50E-03 | 3.59E-01 | 3.37E-02 | 2.56E-02 | 1.77E-05 |
| **IL-18** |  |  |  |  |  |  | 0.00E+00 | 2.19E-01 | 4.97E-01 | 5.40E-01 | 1.09E-01 | 1.23E-01 | 1.48E-01 | 5.12E-01 |
| **MCP1** |  |  |  |  |  |  |  | 0.00E+00 | 8.57E-06 |  | 3.86E-02 | 8.60E-04 | 3.88E-04 | 2.50E-02 |
| **NOS3** |  |  |  |  |  |  |  |  | 0.00E+00 | 1.44E-03 | 4.31E-02 | 9.96E-06 | 8.11E-06 | 1.38E-02 |
| **SOD1** |  |  |  |  |  |  |  |  |  | 0.00E+00 | 1.72E-03 | 1.70E-05 | 1.45E-05 | 4.83E-02 |
| **SOD2** |  |  |  |  |  |  |  |  |  |  | 0.00E+00 | 4.20E-04 | 1.10E-04 | 7.52E-01 |
| **CAT** |  |  |  |  |  |  |  |  |  |  |  | 0.00E+00 | 8.10E-12 | 1.57E-01 |
| **GSTK1** |  |  |  |  |  |  |  |  |  |  |  |  | 0.00E+00 | 1.05E-01 |
| **CDKN2A** |  |  |  |  |  |  |  |  |  |  |  |  |  | 0.00E+00 |

**Table S8. Mean difference of anti-oxidant levels that significantly changed during the controlled human malaria infection. Paired t-test significance level p= 0.05. 95% confidence interval of mean difference is presented as “95% CI”.**

|  | **C-1 /DT+1** | **C-1 / C+64** | **DT+1 /C+64** |
| --- | --- | --- | --- |
| **SOD1** | Mean diff= 0.083 95%CI= 0.017 - 0.148 p= 0.017 | Mean diff.= 0.052 95%CI= -0.006 - 0.111 p= 0.077 | Mean diff.= -0.03 95%CI= -0.112 - 0.051 p= 0.435 |
| **SOD2** | Mean diff.= 0.266 95%CI= 0.151 - 0.381 p< 0.001 | Mean diff.= 0.084 95%CI= -0.002 - 0.17 p= 0.055 | Mean diff.= -0.182 95%CI= -0.34 - -0.024 p= 0.027 |
| **GSTK** | Mean diff.= 0.237 95%CI= 0.19 - 0.283 p< 0.001 | Mean diff.= 0.026 95%CI= -0.028 - 0.081 p= 0.315 | Mean diff.= -0.21 95%CI= -0.284 - -0.136 p< 0.001 |
| **NOS3** | Mean diff.= 0.633 95%CI= 0.525- 0.742 p< 0.001 | Mean diff.= 0.028 95%CI= -0.08 - 0.137 p= 0.584 | Mean diff.= -0.605 95%CI= -0.714 - -0.496 p< 0.001 |
| **CAT** | Mean diff.= 0.457 95%CI= 0.374 - 0.541 p< 0.001 | Mean diff.= 0.028 95%CI= -0.012 - 0.157 p= 0.088 | Mean diff.= -0.385 95%CI= -0.475 - -0.295 p< 0.001 |

**Table S9. Clinical laboratory reference ranges for biochemical liver tests, adapted from Reuling et al.**

|  | **Normal range male** | **Normal range female** |
| --- | --- | --- |
| **AST (U/L)** | <35 | <30 |
| **ALT (U/L)** | <45 | <35 |
| **γGT (U/L)** | <55 | <40 |
| **Alkaline phosphatase (U/L)** | <115 | <100 |
| **Bilirubin (total) (µmol/L)** | <17 | |
| **LD (U/L)** | <250 | |
| **Haemoglobin (mmol/L)** | 8.6-10.5 | 7.5-9.5 |
| **Leukocytes (x10^9^/L)** | 3.5-10.0 | |
| **Lymphocytes (x10^9^/L)** | 0.5-5.0 | |
| **Thrombocytes (x10^9^/L)** | 150-370 | |
| **CRP (mg/L)** | <10 | |
| **Creatinine (µmol/L)** | 65-115 | 55-90 |
| **Urea (mmol/L)** | 2.5-7.5 | |
| **Sodium (mmol/L)** | 136-145 | |
| **Potassium (mmol/L)** | 3.5-5.1 | |
| **Lactate (mmol/L) (plasma)** | 0.5-2.2 | |

**Table S10. Factors of Principle Component Analysis with Eigenvalue >1, explaining collectively 78.59% of the variance in individual characteristics (n = 16, observation = 139).**

| **Number** | **Eigenvalue** | **Percent** | **Cum Percent** | **Chi-Square** | **DF** | **P-value** |
| --- | --- | --- | --- | --- | --- | --- |
| **PCA 1** | 8.10 | 54.02 | 54.02 | 690.09 | 103.89 | < 0.001 |
| **PCA 2** | 2.32 | 15.44 | 69.46 | 377.33 | 103.52 | < 0.001 |
| **PCA 3** | 1.37 | 9.13 | 78.59 | 264.56 | 92.75 | < 0.001 |

**Table S11. Relative contribution of each variable in three significant factors. Grey values show non-significant contribution to each factor.**

|  | **Factor 1** | **Factor 2** | **Factor 3** |
| --- | --- | --- | --- |
| **CDKN2A** | 69.18% | -8.76% | -54.36% |
| **Parasitaemia** | 85.60% | -35.76% | -28.43% |
| **LFT** | 84.21% | -25.53% | -11.23% |
| **IFNγ** | 84.42% | -11.76% | 16.25% |
| **IL8** | 78.59% | -17.39% | -3.24% |
| **IL10** | 84.33% | -35.99% | -19.95% |
| **IL12p70** | 63.59% | -0.84% | 57.59% |
| **IL18** | 65.40% | -14.35% | -18.20% |
| **MCP1** | 82.40% | -15.33% | -12.89% |
| **TEL** | -24.73% | -6.30% | 80.66% |
| **NOS3** | -66.34% | 61.89% | 8.06% |
| **SOD1** | 4.39% | 89.85% | -32.45% |
| **SOD2** | -14.52% | 88.19% | 15.80% |
| **CAT** | -53.90% | 75.04% | 14.35% |
| **GSTK1** | -37.10% | 87.29% | -4.53% |
